# Naringin administration inhibits platelet aggregation and release by reducing blood cholesterol levels and the cytosolic free calcium concentration in hyperlipidemic rabbits

**DOI:** 10.3892/etm.2014.1794

**Published:** 2014-06-20

**Authors:** YANG XIAO, LAI-LAI LI, YAN-YAN WANG, JING-JING GUO, WEN-PING XU, YAN-YAN WANG, YI WANG

**Affiliations:** 1 Institute of Traditional Chinese Medicine Research, Tianjin University of Traditional Chinese Medicine, Tianjin 300193, P.R. China; 2 Tianjin State Key Laboratory of Modern Chinese Medicine, Tianjin University of Traditional Chinese Medicine, Tianjin 300193, P.R. China

**Keywords:** naringin, hyperlipidemic, aggregation, cytosolic free calcium concentration, P-selectin, platelet factor 4

## Abstract

This study investigated the effects of naringin on platelet aggregation and release in hyperlipidemic rabbits, and the underlying mechanisms. The safety of naringin was also investigated. The rabbits were orally administered 60, 30 or 15 mg/kg of naringin once a day for 14 days after being fed a high fat/cholesterol diet for four weeks. Following the two weeks of drug administration, the degree of platelet aggregation induced by arachidonic acid, adenosine diphosphate and collagen was significantly reduced by naringin at certain doses compared with those in the rabbits of the model group (P<0.01). The levels of P-selectin and platelet factor 4 (PF4) also decreased following treatment with naringin compared with those of the model group. Certain doses of naringin significantly reduced the total cholesterol (TC) levels and elevated the ratio of high-density lipoprotein cholesterol to TC compared with those in the model group, and significantly decreased the cytosolic free calcium concentration ([Ca^2+^]_i_). No significant difference in the coagulation function was observed between the control and drug-treatment groups. These results indicate that naringin improved platelet aggregation and inhibited the excessive release of P-selectin and PF4 in hyperlipidemic rabbits. This study suggests that the antiplatelet effect of naringin may be due to its ability to regulate the levels of blood cholesterol and [Ca^2+^]_i_ in platelets. Naringin also did not cause bleeding in the hyperlipidemic rabbits.

## Introduction

Platelets are central mediators of primary homeostasis and mediate pathological thrombosis. Activated platelets stimulate thrombus formation in response to the rupture of an atherosclerotic plaque or endothelial cell erosion, thereby promoting atherothrombotic disease (1). Antiplatelet treatment remains the main therapy for patients with thrombosis and atherosclerosis (2,3). Antiplatelet treatment for the prevention of serious vascular events (including nonfatal myocardial infarction and nonfatal stroke among a large number of patients with a high risk for occlusive vascular events) and vascular death is an important strategy, according to the results of a collaborative meta-analysis of randomized trials (4). However, the number of studies concerning the side-effects attributed to antiplatelet agents (including aspirin, ticlopidine, clopidogrel, abciximab and eptifibatide) is increasing. These negative effects include allergic/hypersensitivity reactions and gastrointestinal disorders, including ulceration of the gastric lining and hemorrhage, as well as increased drug resistance in certain patients (5,6).Studies have shown that various traditional Chinese medicines have antiplatelet activity (7,8). Naringin, a type of flavonoid from Fructus Aurantiin, has numerous biological activities, including anti-inflammatory (9,10) and antioxidant (11,12) activities, regulation of glucose, and lipid metabolism (13–15).

The present study explored the effect of naringin on the aggregation and release of activated platelets in hyperlipidemic rabbits.

## Materials and methods

### Experimental animals

Male New Zealand white rabbits (weight = 2.0–2.5 kg) were provided by Beijing Animal Breeding Center (Beijing, China). The animals were acclimated for at least one week under standard conditions with free access to a standard diet and water. All procedures were approved by the Animal Care and Use Committee of Tianjin University of Traditional Chinese Medicine (approval number: TCM-LAEC2013007; Tianjin, China) and conformed to the Guide for the Care and Use of Laboratory Animals published by the U. S. National Institutes of Health (NIH publication number 85–23, revised 1996).

### Drugs and reagents

Naringin was obtained from Shanghai Meilian Biotechnology Co., Ltd. (Shanghai, China; CAS number: 10236-47-2). Adenosine diphosphate (ADP), arachidonic acid (AA) and collagen (COLL) were purchased from Chrono-Log Corp. (Havertown, PA, USA). ELISA kits for P-selectin and platelet factor 4 (PF4) were obtained from R&D Systems Inc. (Minneapolis, MN, USA). Fibrinogen (FIB), activated partial thromboplastin time (APTT) and prothrombin time (PT) kits were purchased from Stago Diagnosis Technology Co., Ltd. (Paris, France). An intracytoplasmic Ca^2+^ testing kit was obtained from Genmed Scientifics Inc. (Shanghai, China).

### Establishment of hyperlipidemic rabbit model

A total of 30 male rabbits were divided into two groups (control group and group M) according to the total cholesterol (TC) in their plasma. The rabbits in the control group (six males) were fed with a basic diet during the experimental period. The rabbits in group M (24 males) were fed a high fat/cholesterol diet (1% cholesterol, 10% vegetable oil and 89% base animal feeds) for four weeks. The amount of daily diet for each animal was restricted to 50 g, and water was supplied *ad libitum*. After four weeks, blood was collected from the ear edge vein of the rabbits. The 24 hyperlipidemic rabbits in group M were selected based on their significantly higher TC values compared with those of the control group and then were divided into four groups, namely, model group (model), high-dose naringin treatment group (NH; 60 mg/kg/day), medium-dose naringin treatment group (NM; 30 mg/kg/day), and low-dose naringin treatment group (NL; 15 mg/kg/day).

### Drug treatment

The rabbits in the treatment groups were orally administered naringin once a day for 14 consecutive days. The rabbits in the four groups, with the exception of the control group, continued to be fed a high fat/cholesterol diet five times a week to maintain the model.

### Preparation of platelet-rich plasma (PRP) and platelet-poor plasma (PPP)

The rabbits were locally anesthetized with 2% lidocaine (1 ml), and then blood was collected from the common carotid artery (CCA) 2 h after the final drug administration and anticoagulated with citrate (3.8%; 1:9, v/v). PRP was obtained by centrifugation at 800 rpm for 15 min, and the remaining blood was further centrifuged at 3,500 rpm for 10 min to prepare the PPP. The platelet concentration of the PRP was adjusted to 3–5×10^9^ platelets/ml using the PPP.

### Determination of platelet aggregation

Platelet aggregation was measured using an aggregometer (570-VS; Chrono-Log Corp.) according to the methods of Born and Cross (16). In a typical procedure, 0.25 ml PPP and PRP were placed in separate cuvettes and stirred with a rotor at 37°C for 5 min. Platelet aggregation was induced by the addition of ADP, AA or COLL (final concentrations of 13 μM, 500 μM and 10 mg/l, respectively). The results were recorded as light transmission at maximum aggregation following the addition of an aggregating agent. Data are expressed as the percentage maximum aggregation.

### Determination of the FIB levels, PT and APTT

Blood was collected from the CCA and anticoagulated with citrate (3.8%; 1:9, v/v). The plasma was separated by centrifugation at 3,500 rpm for 10 min. The levels of FIB in the plasma, and the PT and APTT were determined with an automatic blood coagulation analyzer (Diagnostica Stago STart 4 hemostasis analyzer; Stago Diagnosis Technology Co., Ltd.)

### Determination of the levels of TC, triglyceride (TG), high-density lipoprotein (HDL) and low-density lipoprotein (LDL)

Blood was collected from the CCA. Following placement in a water bath for 30 min at 37°C, the serum was separated by centrifugation at 3,500 rpm for 15 min. The levels of serum TC, TG, HDL and LDL were determined with an automatic analyzer (7020; Hitachi, Tokyo, Japan).

### Determination of the levels of PF4 and P-selectin

Blood was collected from the CCA. Following placement in a water bath for 30 min at 37°C, the serum was separated by centrifugation at 3,500 rpm for 10 min. The levels of serum P-selectin and PF4 were determined with the ELISA kits according to manufacturer’s instructions.

### Determination of the cytosolic free calcium concentration ([Ca^2+^]_i_)

Following washing twice with Ca^2+^-free Tyrode’s buffer (Beijing Reagan Biotechnology Co., Ltd., Beijing, China), the platelets were suspended in Ca^2+^ Tyrode’s buffer (containing 0.38% bovine serum albumin). The platelet concentration was adjusted to 2×10^8^ platelets/ml, and then [Ca^2+^]_i_ was determined using the intracytoplasmic Ca^2+^ testing kit according to the manufacturer’s instructions.

### Statistical analysis

All data are expressed as the mean ± standard error of the mean. Statistical analysis was performed using analysis of variance. P<0.05 was considered to indicate a statistically significant difference. All statistical analyses were performed using SPSS software, version 11.5 (SPSS, Inc., Chicago, IL, USA).

## Results

### Effects of naringin on the platelet aggregation induced by ADP, AA and COLL

**Figure 1:**
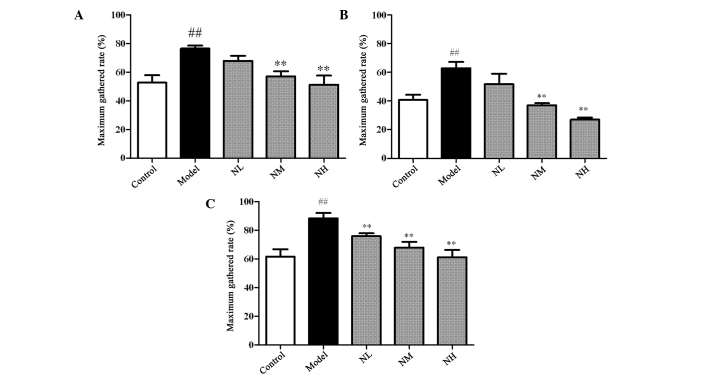
Effects of naringin on (A) AA-, (B) ADP- and (C) COLL-induced platelet aggregation. ^##^P<0.01, compared with the control group; ^**^P<0.01, compared with the model group. NL, low-dose naringin treatment; NM, medium-dose naringin treatment; NH, high-dose naringin treatment; AA, arachidonic acid; ADP, adenosine diphosphate; COLL, collagen.

The maximum gathered rates induced by AA, ADP and COLL in the model group were significantly increased compared with those of the control group (P<0.01), as shown in Fig. 1. The maximum gathered rates induced by AA and ADP were significantly inhibited by the medium and high doses of naringin compared with those of the model group (P<0.01). Each dose of naringin significantly inhibited the maximum gathered rates induced by COLL compared with those of the model group (P<0.01).

### Effects of naringin on the platelet [Ca^2+^]_i_

**Figure 2:**
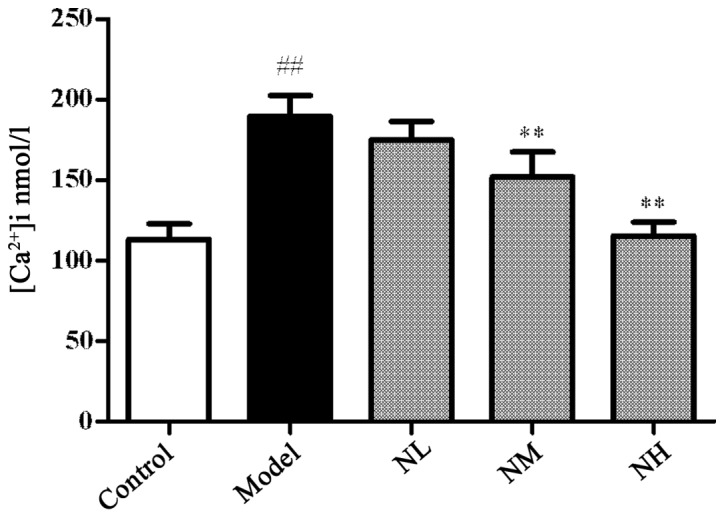
Effects of naringin on the platelet [Ca^2+^]_i_ of the hyperlipidemic rabbits. ^##^P<0.01, compared with the control group; ^**^P<0.01, compared with the model group. [Ca^2+^]_i_, cytosolic free calcium concentration; NL, low-dose naringin treatment; NM, medium-dose naringin treatment; NH, high-dose naringin treatment.

The platelet [Ca^2+^]_i_ significantly increased in the model group compared with that in the control group (P<0.01), and the low dose of naringin had no significant effect on the platelet [Ca^2+^]_i_ compared with that in the model group. However, the medium and high doses of naringin significantly reduced the platelet [Ca^2+^]_i_ compared with that of the model group (P<0.01; Fig. 2).

### Effects of naringin on the levels of P-selectin and PF4 in the hyperlipidemic rabbits

**Figure 3:**
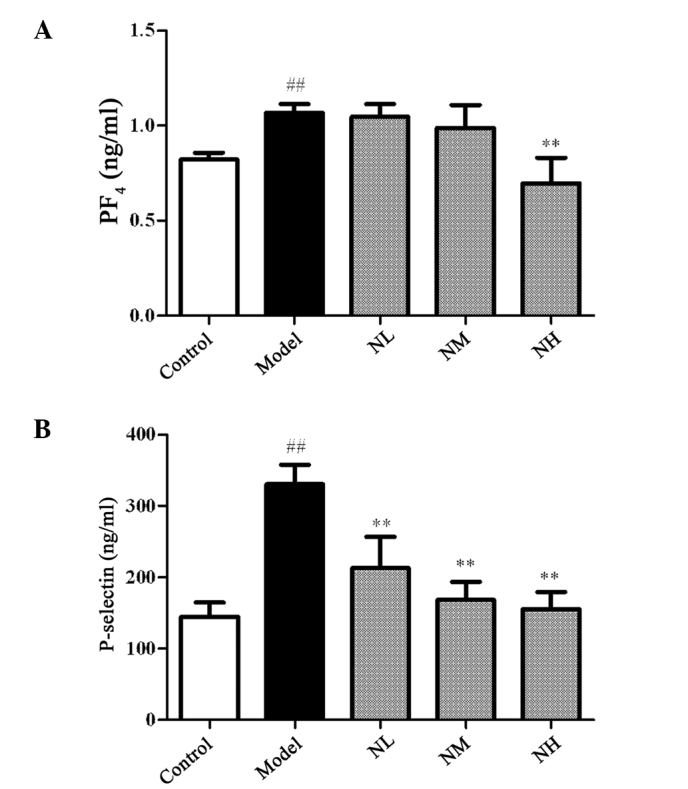
Effects of naringin on the (A) PF4 and (B) P-selectin levels of the hyperlipidemic rabbits. ^##^P<0.01, compared with the control group; ^**^P<0.01, compared with the model group. PF4, platelet factor 4; NL, low-dose naringin treatment; NM, medium-dose naringin treatment; NH, high-dose naringin treatment.

Fig. 3 shows that the levels of PF4 and P-selectin in the model group significantly increased compared with those in the control group (P<0.01). The levels of PF4 were not significantly reduced by the low and medium doses of naringin compared with those in the model group, but were significantly reduced by the high dose (P<0.01). Each dose of naringin significantly reduced the levels of P-selectin in the hyperlipidemic rabbits compared with those in the model group (P<0.01).

### Effects of naringin on the APTT, PT, and FIB levels in the hyperlipidemic rabbits

**Figure 4:**
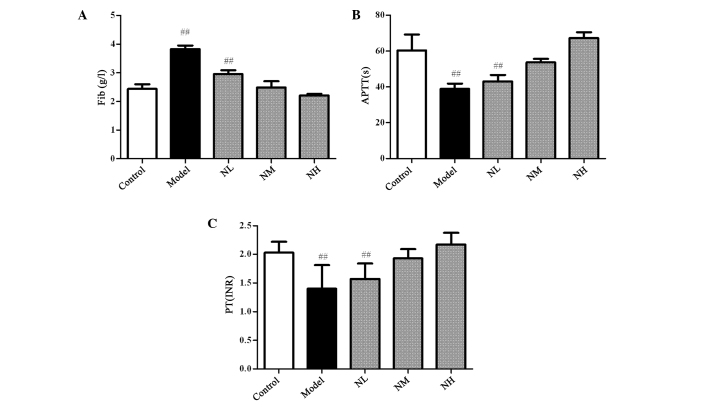
Effects of naringin on the (A) FIB levels, (B) PT and (C) APTT of the hyperlipidemic rabbits. ^##^P<0.01, compared with the control group. FIB, fibrinogen; NL, low-dose naringin treatment; NM, medium-dose naringin treatment; NH, high-dose naringin treatment; APTT, activated partial thromboplastin time; PT, prothrombin time.

In the hyperlipidemic rabbits, the levels of FIB significantly increased, whereas the PT and APTT significantly decreased compared with those of the control group (P<0.01). Naringin reduced the levels of FIB in the plasma and prolonged the PT and APTT to improve the blood hypercoagulable state of the hyperlipidemic rabbit; however, no significant difference was identified in the results for the naringin groups compared with those of the control group (Fig. 4). This indicated that naringin could not cause bleeding.

### Effects of naringin on the levels of blood lipids in the hyperlipidemic rabbits

After four weeks of high-fat feeding, the levels of TC, HDL and LDL significantly increased compared with those in the control group (Table I). However, the ratio of HDL/TC decreased, whereas the ratio of LDL/TC increased compared with those in the control group. The high and medium doses of naringin significantly reduced the levels of TC, HDL and LDL in the plasma, but the HDL/TC ratio significantly increased and the LDL/TC ratio decreased compared with those in the model group (P<0.05-0.01).

## Discussion

Hyperlipidemia reportedly triggers platelet activation, lipid peroxidation, platelet granulocyte aggregation and platelet aggregation. Sener *et al* (17) demonstrated that the expression of P-selectin on the surface of platelets in patients with hyperlipidemia is highly associated with the levels of TG, LDL and HDL-cholesterol. Thus, blood lipids and platelet activation play critical roles in the pathological link of thrombotic diseases to the incidences of other cardiovascular events (18). Naringin has pharmacological activity in the cardiovascular system and plays a role in the regulation of blood glucose and blood lipids. Naringin inhibits the oxidative susceptibility of LDL, which has a certain inhibitory effect on the formation of atherosclerosis (19). The present study focused on the effects of naringin on platelet activation and the coagulation function in the hyperlipidemic pathological state.

The present study demonstrated that naringin exerted positive modulatory effects on the levels of blood lipids in hyperlipemic rabbits. The medium and high doses of naringin significantly decreased the TC levels and increased the proportion of HDL in the TC. Naringin also reduces the sensitivity of platelets by adjusting the levels of blood lipids, thereby achieving the desired effect of antiplatelet aggregation.

As a second messenger, calcium is involved in platelet deformation, aggregation and the release reaction to stimuli. Increased platelet [Ca^2+^]_i_ activates myosin light chain kinase, which leads to platelet deformation and shrinkage. Multiple Ca^2+^-dependent proteases play an important role in platelet activation. Ca^2+^ activates phospholipase C and phospholipase A2, leading to the release of thromboxane A2 and platelets. The activation of protein kinase C, which is involved in the release and aggregation of platelets and AA metabolism, requires the participation of Ca^2+^. The activation state of platelet [Ca^2+^]_i_ in the hyperlipemic animal model in the present study was significantly higher than that in the control group. The results of the present study confirm that naringin reduces the [Ca^2+^]_i_, indicating that naringin inhibits platelet contraction and release by decreasing the platelet [Ca^2+^]_i_ concentration and thereby inhibiting myosin light chain phosphorylation.

Naringin inhibited platelet aggregation induced by AA, ADP and COLL in the present study to appreciable degrees and had a marked dose-dependent effect, indicating that naringin inhibits platelet activation via multiple pathways. The present study also confirmed that naringin had no marked influence on the normal coagulation function in the experimental animals. Further studies are required to elucidate the specific mechanisms of naringin. In conclusion, the results of the present study suggest that naringin inhibits platelet overactivity by regulating the levels of blood lipids and the concentration of platelet cytoplasmic calcium.

## Figures and Tables

**Table I:** Effects of naringin on the blood lipid levels of the hyperlipidemic rabbits.

Group	TC	TG	LDL	HDL	LDL/TC	HDL/TC
Control	0.94±0.164	0.67±0.124	0.22±0.083	0.49±0.097	0.25±0.134	0.55±0.200
Model	11.26±1.283^a^	0.68±0.381	5.50±0.585^a^	2.36±0.317^a^	0.50±0.098^a^	0.21±0.033^a^
NL	9.89±1.989	0.61±0.259	5.11±0.327	2.27±0.390	0.53±0.089	0.24±0.067
NM	6.57±0.594^c^	0.58±0.321	3.46±0.820^c^	1.82±0.472^b^	0.54±0.188	0.27±0.061^b^
NH	3.38±0.260^c^	0.53±0.193	2.02±0.475^c^	1.34±0.399^c^	0.60±0.122	0.40±0.113^c^

TC, total cholesterol; TG, triglyceride; LDL, low-density lipoprotein; HDL, high-density lipoprotein; NL, low-dose naringin treatment; NM, medium-dose naringin treatment; NH, high-dose naringin treatment.

a P<0.01, compared with the control group;

b P<0.05 and

c P<0.01, compared with the model group.
